# Treinamento Físico e Função Endotelial em Hipertensos: Efeitos dos Treinamentos Aeróbico e Resistido

**DOI:** 10.36660/abc.20210111

**Published:** 2021-05-06

**Affiliations:** 1 Universidade de São Paulo Escola de Artes, Ciências e Humanidades São PauloSP Brasil Escola de Artes, Ciências e Humanidades da Universidade de São Paulo, São Paulo, SP - Brasil; 2 Universidade de São Paulo Escola de Educação Física e Esporte Laboratório de Hemodinâmica da Atividade Motora São PauloSP Brasil Laboratório de Hemodinâmica da Atividade Motora, Escola de Educação Física e Esporte da Universidade de São Paulo, São Paulo, SP - Brasil; 3 Universidade de São Paulo Grupo de Pesquisa em Cronobiologia Aplicada & Fisiologia do Exercício São PauloSP Brasil Grupo de Pesquisa em Cronobiologia Aplicada & Fisiologia do Exercício, Universidade de São Paulo, São Paulo, SP - Brasil

**Keywords:** Exercício Físico, Treino Aeróbico, Resistência Física, Hipertensão, Endotélio Vascular

A hipertensão é um dos fatores de risco mais importantes para eventos cardiovasculares, e está fortemente relacionada à disfunção endotelial^1^ A função endotelial avaliada pela técnica de dilatação mediada por fluxo (DMF) é 3,2% menor em hipertensos do que em normotensos.^2^ O balanço negativo entre dano e regeneração de células endoteliais, indicado por um elevado número de micropartículas endoteliais (MPE) circulantes e níveis reduzidos de células progenitoras endoteliais (CPE), é preditor de eventos cardiovasculares em hipertensos.^3^ Assim, terapias capazes de influenciar positivamente a função endotelial são importantes para melhorar o prognóstico na hipertensão.

A prática regular de exercícios é recomendada não apenas pelo benefício de reduzir a pressão arterial, mas também para reduzir a morbidade e mortalidade cardiovascular em hipertensos.^4^ Importantes adaptações vasculares induzidas por exercício, principalmente resultando em aumento da vasodilatação dependente do endotélio, podem explicar parcialmente esses benefícios do exercício. Uma meta-análise prévia mostrou efeitos positivos do exercício aeróbico na função endotelial de hipertensos.^5^ No entanto, a variação dos resultados sugere que diferentes protocolos de exercício (tipos e intensidade) e características dos participantes podem influenciar a resposta da função endotelial ao treinamento físico. Além disso, é necessário saber os mecanismos subjacentes para a melhora endotelial.

Diante disso, Waclawovsky et al.,^6^ ajudaram a esclarecer alguns desses pontos. Os autores pesquisaram a literatura em busca de ensaios clínicos randomizados (ECR) que investigassem os efeitos de diferentes protocolos de treinamento físico na função endotelial, MPE e CPE em pré-hipertensos e hipertensos. Foram pesquisadas diferentes bases de dados (e.g.: MEDLINE, Cochrane, LILACS, EMBASE e SciELO), e aplicou-se a estratégia PICOS para conseguir 10 estudos elegíveis.

Em relação ao exercício aeróbico, 9 estudos envolveram grupos de treinamento aeróbico permitindo especulações de dose-resposta em relação à melhora vascular. Assim, os autores sugeriram que o treinamento de intensidade moderada realizado 3 vezes/semana por 30–40 min pode ser melhor para melhorar a função endotelial em hipertensos, enquanto o treinamento intervalado vigoroso pode ser uma alternativa em pré-hipertensos. Na verdade, um estudo anterior detectou que cada aumento absoluto (2-MET) ou relativo (10%) na intensidade do treinamento aeróbico resulta em uma melhora de quase 1% na função endotelial sem influência do volume de treinamento.^7^ A teoria subjacente aos maiores benefícios propiciados pela maior intensidade se baseia na maior taxa de cisalhamento produzida por um fluxo sanguíneo mais rápido levando a níveis mais elevados de óxido nítrico. Digno de nota, um estudo de Waclawovsky et al.,^6^ avaliou hipertensos sem síndrome metabólica ou doença cardiovascular, demonstrando benefício específico do treinamento aeróbico para hipertensão, independente de comorbidades. Estudos anteriores envolvendo diferentes populações (por exemplo: saudáveis, hipertensos, diabéticos, pacientes cardíacos, etc.) sugerem que as características antropométricas e de saúde podem influenciar as melhorias da função endotelial induzidas pelo treinamento aeróbico. De fato, análises de subgrupo em uma meta-análise anterior demonstraram que indivíduos não obesos apresentam maior melhora da função endotelial com treinamento aeróbico do que obesos. Além disso, indivíduos com menores valores basais de DMF apresentam mais melhorias após o treinamento do que aqueles que apresentam valores basais mais elevados.^7^

Em relação ao exercício resistido, Waclawovsky et al.,^6^ encontraram apenas dois estudos que incluíram grupos de exercícios resistidos dinâmicos e ambos apresentaram resultados de função endotelial positivos. No entanto, um estudo publicado após a busca dos autores (2020) não encontrou nenhuma alteração na função endotelial com esse tipo de treino em comparação com o grupo controle.^8^ Além disso, a revisão de Waclawovsky et al.,^6^ encontrou o único estudo com exercício isométrico resistido, que aumentou a DMF, mas apenas em braços treinados,^9^ sugerindo um efeito local desse tipo de exercício. Portanto, com base no pequeno número de estudos, qualquer conclusão sobre os efeitos do exercício resistido na função endotelial na hipertensão ou seus fatores de influência é arriscada. No entanto, uma revisão com populações mais abrangentes, incluindo hipertensos, revelou um efeito positivo do exercício dinâmico de resistência na função endotelial.^7^

Em relação aos mecanismos de melhora da função endotelial induzida pelo exercício, espera-se o aumento da biodisponibilidade do óxido nítrico por meio da redução de sua degradação por radicais livres.^10^ Além disso, o equilíbrio entre o dano e a regeneração das células endoteliais surgiu como uma ferramenta promissora. Relatou-se a redução das MPE e um aumento das CPE (isto é, biomarcador de reparo) após o exercício físico em amostras heterogêneas.^11^^,^^12^ No entanto, Waclawovsky et al.,^6^ não conseguiram encontrar nenhum ECR que investigasse os efeitos de qualquer tipo de exercício físico sobre esses biomarcadores em hipertensos. Isso revelou uma importante lacuna na literatura e a necessidade de estudos futuros.

Em resumo, os achados relatados por Waclawovsky et al.,^6^ contribuem para a literatura ao confirmar o efeito positivo do treinamento aeróbico na melhora da função endotelial em hipertensos. Também sugere que a intensidade do exercício aeróbico pode influenciar essa melhora, o que deve ser reforçado com mais estudos. No entanto, o falta de estudos com exercício resistido dinâmico e isométrico em hipertensos expôs a necessidade de mais ECRs para permitir conclusões robustas sobre seu benefício na função endotelial. Finalmente, a literatura sugere que o equilíbrio entre dano e regeneração do tecido endotelial parece ser uma chave promissora para entender os benefícios do exercício na função endotelial. Waclawovsky et al.,^6^ mostrou claramente a necessidade de estudos que investiguem os efeitos do exercício sobre os mecanismos da função endotelial.
