# Supplementary material for: Treinamento Físico e Função Endotelial em Hipertensos: Efeitos dos Treinamentos Aeróbico e Resistido
Source: Arq Bras Cardiol. 2021 May 6;116(5):948–9. [Article in Portuguese] doi: 10.36660/abc.20210111 (PMC8121457; doi:10.36660/abc.20210111)
Supplement: Translation

# Translation: Exercise Training and Endothelial Function in Hypertension: Effects of Aerobic and Resistance Training

**Affiliations:** 1 São PauloSP Brazil Escola de Artes, Ciências e Humanidades da Universidade de São Paulo, São Paulo, SP - Brazil; 2 São PauloSP Brazil Laboratório de Hemodinâmica da Atividade Motora, Escola de Educação Física e Esporte da Universidade de São Paulo, São Paulo, SP - Brazil; 3 São PauloSP Brazil Grupo de Pesquisa em Cronobiologia Aplicada & Fisiologia do Exercício, Universidade de São Paulo, São Paulo, SP - Brazil

**Keywords:** Exercise, Endurance Training, Physical Resistance, Hypertension, Endothelium Vascular

Hypertension is one of the most important risk factors for cardiovascular events, which is strongly related with endothelial dysfunction.^1^ Endothelial function assessed by flow-mediated dilation (FMD) is 3.2% lower in hypertensives than in normotensives.^2^ The negative balance between damage and regeneration of endothelial cells indicated by a high number of circulating endothelial microparticles (EMP) and reduced levels of endothelial progenitor cells (EPC) is predictor of cardiovascular events in hypertensives.^3^ Thus, therapies able to positively influence endothelial function are important to improve prognosis in hypertension.

Regular practice of exercise is recommended not only for the benefit of lowering blood pressure, but also for reducing cardiovascular morbidity and mortality in hypertensives.^4^ Important exercise-induced vascular adaptations, mainly resulting in increased endothelium-dependent vasodilation, may partially explain these exercise benefits. A previous meta-analysis showed positive effects of aerobic exercise on the endothelial function of hypertensives.^5^ However, the variation of results suggests that different exercise protocols (types and intensity) and subjects’ characteristics may influence the response of endothelial function to exercise training. In addition, the underlying mechanisms for endothelial improvement need to be known.

Given that, Waclawovsky et al.^6^ helped to clarify some of these points. The authors searched the literature looking for randomized controlled trials (RCT) investigating the effects of different exercise training protocols on endothelial function, EPM and EPC in pre-hypertensives and hypertensives. Different databases (e.g.: MEDLINE, Cochrane, LILACS, EMBASE and SciELO) were searched and the PICOS strategy was applied to reach 10 eligible studies.

Regarding aerobic exercise, 9 studies involved aerobic training groups allowing for dose-response speculations regarding vascular improvement. Thus, the authors suggested that moderate intensity training performed 3 times/week for 30–40 min may be better to improve endothelial function in hypertensives, while vigorous interval training might be an alternative in pre-hypertensives. Actually, a previous study detected that every absolute (2-MET) or relative (10 %) increase in the intensity of aerobic training results in nearly 1% improvement in endothelial function with no influence from volume of training.^7^ Theory behind higher intensity promoting greater benefits relies on the greater shear rate produced by faster blood flow leading to higher nitric oxide levels. Noteworthy, a study by Waclawovsky et al.^6^ evaluated hypertensives without metabolic syndrome or cardiovascular disease, showing a specific benefit of aerobic training for hypertension regardless of comorbidities. Previous studies involving different populations (e.g.: healthy, hypertensives, diabetics, cardiac patients, etc.) suggest that health and anthropometric characteristics may influence endothelial function improvements induced by aerobic training. Indeed, subgroup analyses in a previous meta-analysis demonstrated that non-obese individuals present greater enhancement in endothelial function with aerobic training than obese ones. Additionally, individuals with lower baseline FMD values present greater improvements after training than those who present higher baseline values.^7^

Concerning resistance exercise, Waclawovsky et al.^6^ found only two studies that included dynamic resistance exercise groups and both presented positive endothelial function results. However, a study published after the authors’ search (2020) did not find any change in endothelial function with this type of training compared with the control group.^8^ Additionally, Waclawovsky et al.'s review^6^ found the only one study with isometric resistance training and this training increased FMD but only in trained arms,^9^ suggesting a local effect of this type of exercise. Therefore, based on the few number of studies, any conclusion about the effects of resistance training on endothelial function in hypertension or its factors of influence is risky. Nevertheless, a review with more comprehensive populations, including hypertensives, revealed a positive effect of dynamic resistance training on endothelial function.^7^

Regarding the mechanisms for exercise-induced endothelial function improvement, the enhancement of nitric oxide bioavailability by reducing its degradation by free radicals is expected.^10^ In addition, the balance between damaging and regeneration of endothelial cells has emerged as a promising tool. The reduction of EMP and an increase of EPC (i.e. repair biomarker) has been reported after exercise training in heterogeneous samples.^11^^,^^12^ However, Waclawovsky et al.^6^ were not able to find any RCTs investigating the effects of any type of exercise training on these biomarkers in hypertensives. This revealed an important lack in the literature and the need for future studies.

In summary, the findings reported by Waclawovsky et al.^6^ contribute to the literature by confirming the positive effect of aerobic training improving endothelial function in hypertensives. It also suggests that the intensity of aerobic exercise may influence this improvement, which should be strengthened with more studies. However, the lack of studies with dynamic and isometric resistance training in hypertensives exposed the need for more RCTs to allow robust conclusions about their benefit on endothelial function. Finally, the literature suggests that balance between damage and regeneration of endothelial tissue seems to be a promising key to understand exercise benefits in endothelial function. Waclawovsky et al.^6^ clearly showed the need for studies investigating the exercise effects on endothelial function mechanisms.
